# Magnetic-Resonance Diffusion-Tensor Tractography in the Diagnosis of Tumefactive Spinal-Cord Lesions in Neuromyelitis Optica

**DOI:** 10.3390/diagnostics10060401

**Published:** 2020-06-12

**Authors:** Yung Hsu, Ming-Chung Chou, Poh-Shiow Yeh, Te-Chang Wu, Ching-Chung Ko, Tai-Yuan Chen

**Affiliations:** 1 Department of Medical Imaging, Chi Mei Medical Center, Tainan 710, Taiwan; lewdster0128@gmail.com (Y.H.); porthoswu@yahoo.com.tw (T.-C.W.); crazyboy0729@gmail.com (C.-C.K.); 2 Department of Medical Imaging and Radiological Sciences, College of Health Sciences, Kaohsiung Medical University, Kaohsiung 807, Taiwan; mcchou@kmu.edu.tw; 3 Department of Medical Research, Kaohsiung Medical University Hospital, Kaohsiung 807, Taiwan; 4 Center for Big Data Research, Kaohsiung Medical University, Kaohsiung 807, Taiwan; 5 Department of Neurology, Chi Mei Medical Center, Tainan 710, Taiwan; poh.shiow@msa.hinet.net; 6 Department of Neurology, Taipei Medical University, Taipei 110, Taiwan; 7 Department of Medical Sciences Industry, Chang Jung Christian University, Tainan 711, Taiwan; 8 Department of Biomedical Imaging and Radiological Sciences, National Yang-Ming University, Taipei 112, Taiwan; 9 Department of Pharmacy, Chia Nan University of Pharmacy and Science, Tainan 717, Taiwan; 10 Graduate Institute of Medical Sciences, Chang Jung Christian University, Tainan 711, Taiwan

**Keywords:** magnetic-resonance imaging, diffusion-tensor imaging, diffusion-tensor tractography, tumefactive, spinal cord, neuromyelitis optica

## Abstract

Magnetic-resonance (MR) imaging is the modality of choice for the evaluation of spinal-cord lesions. However, challenges persist in discriminating demyelinating processes from neoplastic lesions using conventional MR sequences. Consequently, an invasive spinal-cord biopsy is likely for most patients. MR diffusion-tensor imaging is an emerging noninvasive and powerful method for characterizing changes in tissue microstructure associated with spinal disorders. We currently present the case of a middle-aged woman suffering from neuromyelitis optica, and highlight that MR diffusion-tensor tractography can be helpful in the identification of tumefactive spinal-cord lesions.

## 1. Introduction

Cervical myelopathy is a common preliminary diagnosis with variable and confusing clinical symptoms and signs that can be attributed to tumors, demyelination, inflammation, degeneration, mechanical injury, infection, vascular lesions, or vascular insults [1]. Magnetic-resonance imaging (MRI) is the modality of choice for the diagnosis of spinal-cord abnormalities. Intramedullary lesions are typically approached using conventional MRI with emphasis on the location and length of segment involvement, cross-sectional distribution, and an enhancement pattern that aims to narrow differential diagnosis and guide-appropriate management [1,2]. However, discriminating intramedullary non-neoplastic lesions from tumors remains challenging. After spinal-cord biopsy, up to 16% of suspected intramedullary tumors were proven to be demyelinating lesions [3,4]. Therefore, spinal-cord biopsy, an invasive procedure with higher potential risk of neurological deficits, is still highly likely even if the diagnosis of a tumor is not excluded.

Diffusion-weighted imaging (DWI) and diffusion-tensor imaging (DTI) are advanced MRI techniques conducted by measuring the Brownian motion of water molecules within a voxel of tissue [5]. DWI shows the magnitude of the diffusion, irrespective of directional dependence, by referring to the actual apparent diffusion-coefficient (ADC) value [6]. DTI has been utilized to estimate three-dimensional distribution of water diffusivities (λ1, λ2, λ3) in vivo, from which axial (AD), radial (RD), and mean diffusivity (MD), and fractional anisotropy (FA) can be calculated. AD (λ1) and RD ((λ2 + λ3)/2) are diffusivities measured in parallel and perpendicular to the principal axis of the diffusion tensors, respectively. MD ((λ1 + λ2 + λ3)/3) is the averaged diffusivity of a diffusion tensor. FA values range from zero (perfect isotropy) to one (progressive anisotropy). On the basis of the principal diffusion direction of a diffusion tensor, the probable path of white-matter (WM) tracts, but not real axonal tracts, could be reconstructed in a process known as diffusion-tensor tractography (DTT) [7].

DTI could provide additional insights into spinal microstructures. DTI metrics may correspond to microstructural changes and pathological information. Among them, FA reflects anisotropic diffusion and is an index of tissue integrity, AD and RD may be useful surrogate markers of axonal and myelin damage [8], and MD is sensitive to cellularity, edema, and necrosis [9]. Previous studies demonstrated that intramedullary neoplasm has lower FA values when using a cut-off point of 0.272, but there is still some debate [10,11]. DTT is now commonly used in the brain, but is less commonly used in the spinal cord despite it being a highly anisotropic structure suitable for DTI study owing to its small size, being surrounded by vertebral bony elements, and having physiologic motions [12,13]. We present a case that utilized MR DTI metrics and DTT to assist in the diagnosis of a tumefactive spinal-cord lesion in neuromyelitis optica (NMO). Informed consent was obtained from the patient.

## 2. Case Report

**Figure 1:**
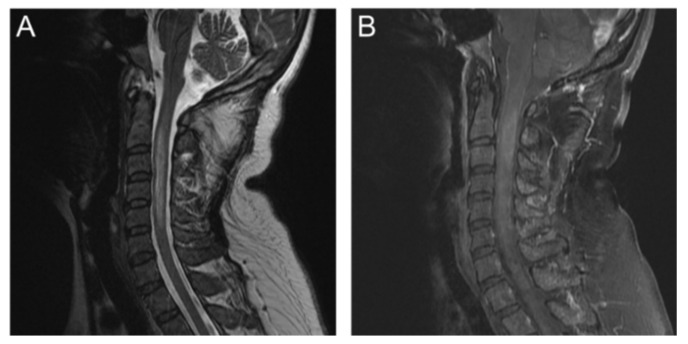
Initial conventional magnetic-resonance imaging MRI. (**A**) T2-weighted image (T2WI) showed mild spinal-cord swelling with hyperintensity lesion in C2–C5 spinal cord; (**B**) postcontrast T1-weighted image (T1WI) with fat suppression showed intramedullary lesion with faint enhancement in C2–C3 spinal cord.

A 50-year-old female reported progressive numbness and weakness of her right limbs without remarkable medical history or trauma during a visit to the emergency room. Her consciousness was clear without evident abnormalities in muscle tone, reflex, gait, or sphincter function. The muscle strength of her right limbs was 4/5, and the sensory level was C4. Laboratory tests revealed elevated an aspartate aminotransferase (AST) level of 144 U/L, an alanine aminotransferase (ALT) level of 67 U/L and a glycated hemoglobin (HbA1c) level of 6.6%, but other levels were unremarkable. Initial brain MRI revealed nonspecific intracranial findings, but showed an intramedullary lesion in the upper cervical spinal cord. Subsequent cervical MRI showed a faintly enhanced infiltrative lesion at the right posterior aspect of the spinal cord at C2 to C3 with extensive edema at C2 through C5 (Figure 1). Due to the impression of C2–C3 intramedullary tumor with the deterioration of neurological symptoms, she received spinal decompressive surgery. A frozen section of an intraoperative biopsy was suggestive of a low-grade glial neoplasm. The weakness of her right limbs improved after the operation.

**Figure 2:**
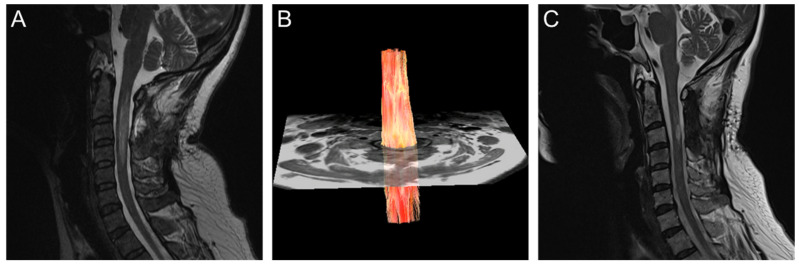
Subsequent MRI study. (**A**) T2-weighted image (T2WI) showed persistent hyperintensity lesion in C2–C4 spinal cord; (**B**) diffusion-tensor tractography (DTT) showed intact streamline of white-matter tracts without displacement or interruption; (**C**) T2WI after 2 months of medical treatment showed residual faint hyperintensity in C2 spinal cord, and focal myelomalacia corresponding to prior excision biopsy in C3 spinal cord.

**Figure 3:**
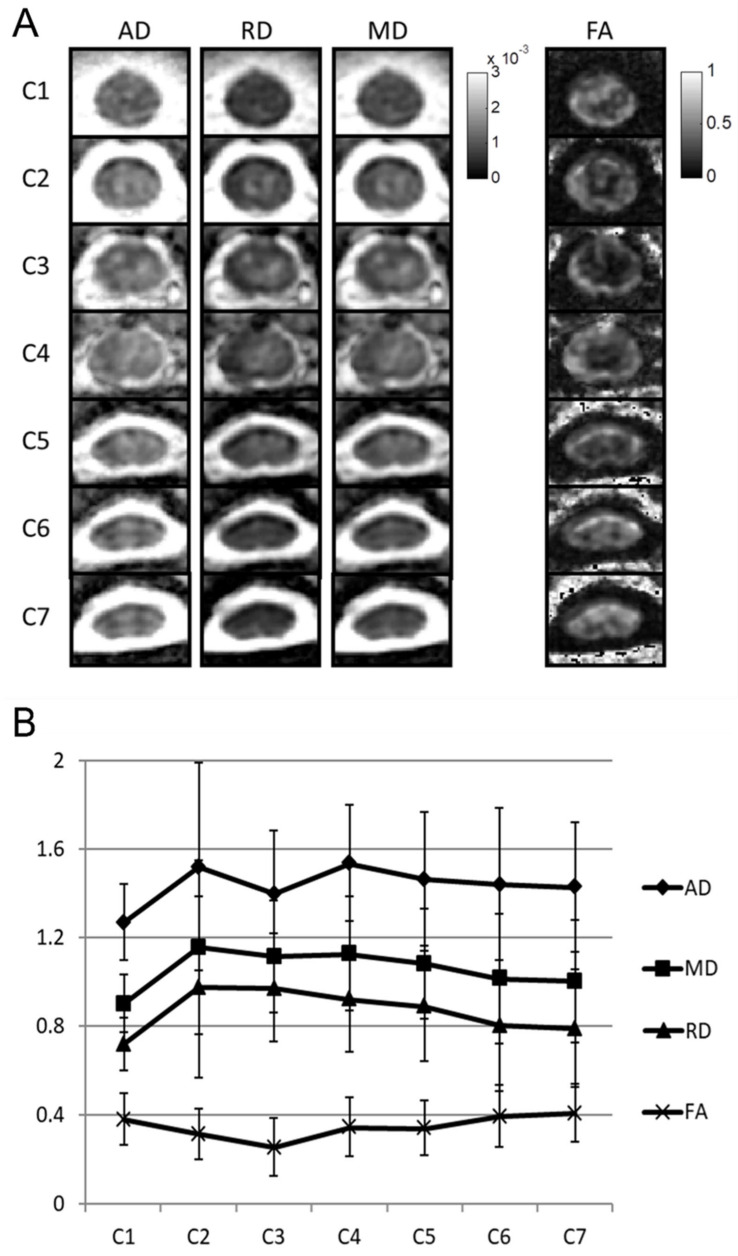
Diffusion-tensor-imaging (DTI) analysis of spinal cord; (**A**) axial diffusivity (AD), radial diffusivity (RD), mean diffusivity (MD), and fractional-anisotropy (FA) maps at levels from C1 to C7; (**B**) mean and standard deviation of four DTI metrics at levels from C1 to C7. Unit for AD, RD, and MD is 10^−3^ mm^2^/s. FA is dimensionless with a range between 0 and 1.

Subsequent pathology of the permanent specimen revealed a histiocytic lesion that was suggestive of an inflammatory demyelinating process or Erdheim–Chester disease. Her visual evoked potential test results were unremarkable. Oral prednisolone of 5 mg/day was initially prescribed, but she then developed weakness in her left limbs. Follow-up cervical MRI showed a persistent C2–C4 intramedullary lesion (Figure 2A) as did DTI (GE, DISCOVERY MR750 3.0 Tesla scanner; protocol: single-shot echo-planar imaging, TR: 3000 ms, TE: 66 ms, flip angle: 90 degrees, array coil spatial-sensitivity encoding factor of 2, b-values of 0 and 600 s/mm^2^, 9 noncollinear diffusion-gradient directions, field of view of 100 × 100 mm^2^, matrix size of 96 × 48, axial slice thickness of 5 mm) with reconstruction of fiber tractography (FT) using deterministic FiberTrak algorithms of FuncTool in a vendor workstation. FT results showed a preserved streamline of WM tracts without displacement or interruption (Figure 2B). DTI analysis of the spinal cord lesions also showed decreased FA (0.258 ± 0.128) and AD values (1.401 ± 0.284 × 10^−3^ mm^2^/s), but increased RD (0.975 ± 0.254 × 10^−3^ mm^2^/s) and MD values (1.117 ± 0.244 × 10^−3^ mm^2^/s) at the C3 level as compared with the nonlesion spinal cord at the C1 and C7 levels (Figure 3). These DTI metrics and DTT findings were suggestive of inflammatory demyelinating processes, such as multiple sclerosis or NMO spectrum disorders, rather than an intramedullary tumor. Eventually, the diagnosis of NMO spectrum disorders was established on the basis of the 2015 International Panel for NMO Spectrum Disorders Diagnosis criteria and a positive test for aquaporin-4 (AQP-4) IgG antibodies [14]. Symptoms of weakness in her limbs improved gradually after intravenous steroid pulse therapy (methylprednisolone, 1000 mg for 5 days) followed by oral immunosuppressants (azathioprine, 25 mg per day). After 2 months of medical treatment, follow-up MRI showed marked regression of the cervical spinal-cord lesion with focal myelomalacia corresponding to the prior excision biopsy at the C3 spinal cord (Figure 2C).

## 3. Discussion

**Figure 4:**
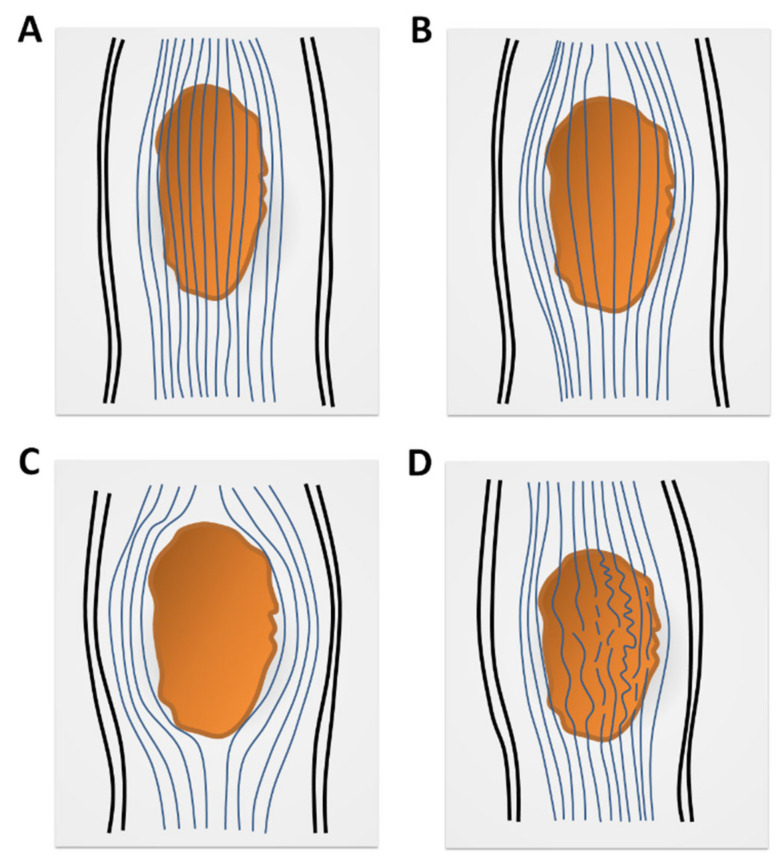
Schematic images of diffusion-tensor-tractography (DTT) features in spinal-cord lesions (the orange areas in the images). (**A**) Intact white-matter tract; (**B**) spread of white-matter tracts with fibers entering lesion; (**C**) displaced white-matter tracts without fibers entering lesion; (**D**) interrupted white-matter tracts. Illustrations derived and modified from Liu X et al. [16].

Spinal DTI is gaining acceptance by overcoming associated technical challenges by using scanners with higher Tesla for better signal-to-noise ratio, faster imaging sequences for fewer motion artefacts, lower B values for fewer susceptibility artefacts, more diffusion-gradient directions, a smaller field of view, smaller reconstructive matrix, and smaller slice thickness than what has been used in brain DTI [15,16]. Therefore, spinal DTT could be reconstructed to demonstrate the streamline of WM tracts on the basis of DTI analysis. According to previous studies, the streamline of spinal WM tracts could present as intact, spreading, displaced, or interrupted (Figure 4) [17,18,19]. Renoux et al. conducted a DTI study on 15 patients with myelitis, and results showed that DTT presented as spreading or interrupted WM tracts [17]. Spinal DTT may show the interrupted or displaced streamline of WM tracts in astrocytoma, and the displacement of WM tracts in ependymoma [16]. Egger et al., and Mohamed et al. performed a spinal DTT study that demonstrated inflammatory demyelinating processes could show the intact or nondisplaced streamline of WM tracts, but neoplastic processes always presented as the displaced or interrupted streamline of WM tracts [18,19].

NMO spectrum disorders are autoimmune demyelinating disorders that are associated with axonal loss, perivascular lymphocytic infiltration, and vascular proliferation [20]. Abnormal MR signals usually involve sites that highly express AQP-4 antigens. These include circumventricular organs in the brain, optic nerves/chiasm, and central gray matter along the central canal of the spinal cord, which usually show longitudinally extensive transverse myelitis (LETM) [21]. In the acute phase of NMO, spinal-cord swelling with irregular enhancement may mimic intramedullary neoplasm [21]. In the current case, the patient presented with spinal-cord LETM without involved lesions in the brain or optic nerves, which prompted an initial consideration of intramedullary neoplasm. However, a subsequent MR DTT study showed a normal streamline of WM tracts that favored inflammatory demyelinating processes, which was consistent with the pathological and clinical diagnosis of NMO spectrum disorders rather than intramedullary neoplasm.

The present study also employed DTI metrics to characterize tissue alterations of spinal-cord lesions. FA values for a normal cervical spinal cord were reported to be between 0.6 and 0.7 [22,23,24]. In the current case, the lowest FA value of the spinal cord was 0.258 ± 0.128 at the C3 level, associated with decreased values in AD (1.401 ± 0.284 × 10^−3^ mm^2^/s), as well as increased RD (0.975 ± 0.254 × 10^−3^ mm^2^/s) and MD (1.117 ± 0.244 × 10^−3^ mm^2^/s), as compared with normal values for the spinal cord at the C3 level [24]. Previous studies using mice found that axonal injury resulted in decreased AD, while demyelination led to increased RD [8,25]. The increased MD could be attributed to vasogenic edema [26]. During inflammation, where the infiltration of cells coexists with axonal injury and demyelination, DTI may underestimate the degree of demyelination (less increase in RD) and overestimate the degree of axonal injury (greater decrease in AD) [27]. Consistent with a previous study examining DTI [28], results in the current study demonstrated that NMO led to increased RD and MD, but decreased AD and FA values in the lesions. In addition, the percentage change of RD (at C2–C4 levels) was more prominent than other DTI metrics. In the early phase of NMO, initial inflammatory changes may cause diminished AD, and subsequent demyelination may be characterized by increased RD [29]. However, in chronic diseases with intensive axonal loss, which causes diminished tissue directionality, it leads to increased AD, RD, and MD [29]. Therefore, the timing of inflammation may result in changes of DTI indices that may be different. Multiple other factors support the influence of coexistent edema, cell infiltration, axonal injury/loss, and demyelination. In the present case, more prominently increased RD than decreased AD could suggest that the pathology of lesions was dominated by a demyelinating process, and partially affected by vasogenic edema.

Some limitations warrant discussion. First, DTI data were only acquired from the patient after treatment, so we were unable to quantify the percentage change of microstructural diffusion in NMO spinal lesions after the treatment. Second, this study only reported a patient with NMO spinal lesions. A study enrolling more patients with NMO spinal lesions is suggested for the future. Finally, this study did not compare DTI and DTT results between patients with demyelinating pathology and neoplasm in the spinal cord. Therefore, further investigations are needed to better understand the differences of microstructural diffusion changes between them.

## 4. Conclusions

In conclusion, MR DTI can provide additional insights into the pathophysiology and microstructural changes of NMO spinal lesions. DTT could provide the treating physician with information for a more convincing judgment when discriminating spinal inflammatory demyelinating processes from intramedullary neoplasm. Intact streamline of WM tracts without displacement or interruption is highly suggestive of non-neoplastic processes. Diagnosis based on clinical information, imaging findings, and response of medical treatment prior to spinal-cord biopsy could be attempted in an adequate clinical situation.
